# Supplementary material for: Possible Association of Energy Availability with Transferrin Saturation and Serum Iron during Summer Camp in Male Collegiate Rugby Players
Source: Nutrients. 2021 Aug 26;13(9):2963. doi: 10.3390/nu13092963 (PMC8466518; doi:10.3390/nu13092963)
Supplement: Supplementary file 1 [file nutrients-13-02963-s001.zip › nutrients-1314655-supplementary.pdf]

Supplementary

Table S1. Biochemical and hematological parameters during the training camp.

	Reference Range		Position	n	Pre		Mid			Post		
					EMM	SE	EMM	SE		EMM	SE	
ALB	3.8–5.3	g/dL	T5	7	4.7	0.1	4.7	0.1		4.7	< 0.1	
			BR	4	4.7	< 0.1	4.8	< 0.1	**	4.6	0.1	#
			BK	8	4.6	0.1	4.7	< 0.1		4.6	0.1	
CK	50–250	U/L	T5	7	811.6	173.2	758.0	108.5		411.3	87.2	**, ###
			BR	4	1834.3	305.6	1233.8	186.9		659.3	147.6	***, ###
			BK	8	315.1	86.7	802.5	101.7	***	436.9	53.8	***, ###
Hepcidin	79.0–49,000	pg/mL	T5	7	23,651.2	4454.6	17,755.4	1843.5		15,556.4	2642.0	
			BR	4	22,465.9	3199.9	17,994.0	1636.9		13,715.6	1755.2	
			BK	8	10,776.7	1747.1	12,821.0	714.7		12,343.8	2331.6	
WBC	3900–9800	/μL	T5	7	5957.1	384.3	6528.6	664.8	**	5942.9	578.5	*
			BR	4	6875.0	89.3	6425.0	138.6		6025.0	460.1	
			BK	8	5625.0	284.9	6062.5	360.5	*	6162.5	526.8	
RBC	427–570	$\times 10^4$/μL	T5	7	512.3	9.1	510.1	12.4		514.6	8.4	
			BR	4	487.3	5.6	499.3	7.7	***	497.0	7.5	**, #
			BK	8	512.8	12.3	509.0	12.8		510.9	13.4	
Hb	13.5–17.6	g/dL	T5	7	15.1	0.3	15.1	0.2		15.3	0.1	
			BR	4	14.9	0.4	15.1	0.4	*	15.0	0.5	**
			BK	8	15.5	0.3	15.4	0.3		15.6	0.4	
Hct	39.8–51.8	%	T5	7	46.8	1.1	46.5	0.7		48.3	0.4	##
			BR	4	45.4	0.8	46.2	1.1	**	47.2	1.3	**, #
			BK	8	48.3	0.8	46.9	0.6	***	48.4	1.0	#
MCV	83–102	fL	T5	7	91.6	1.6	91.1	1.5		94.0	1.4	***, ###
			BR	4	93.5	1.1	92.8	1.1	***	95.0	1.1	##
			BK	8	94.4	1.0	92.3	1.3	***	95.0	1.1	*, ###
MCH	28.0–34.6	pg	T5	7	29.5	0.5	29.6	0.4		29.8	0.5	
			BR	4	30.5	0.6	30.1	0.6		30.2	0.6	**
			BK	8	30.2	0.4	30.3	0.4		30.5	0.3	*
MCHC	31.6–36.6	%	T5	7	32.3	0.2	32.4	0.2		31.7	0.2	**, ###
			BR	4	32.7	0.3	32.6	0.3		31.8	0.4	**, ##
			BK	8	32.1	0.2	32.8	0.2	***	32.1	0.2	###
PLT	13.0–36.9	$\times 10^4$/μL	T5	7	25.3	1.3	26.2	1.5		26.3	1.7	
			BR	4	28.4	1.7	28.2	1.9		27.3	1.4	*
			BK	8	23.5	1.4	22.9	2.0		25.7	1.0	**, #

EMM estimated marginal mean; SE standard error; * $p < 0.05$, ** $p < 0.01$, *** $p < 0.001$ vs. Pre; # $p < 0.05$, ## $p < 0.01$, ### $p < 0.001$ vs. Mid; T5, Tight-5 (numbers 1–5); BR, Back row (numbers 6–8); and BK, Backs (numbers 9–15).

Supplementary

Table S2. Physical activity in the first half of the pre-season camp.

(**A**) Forwards

	Category	CODE	METs	8/9		8/10		8/11		8/12		8/13		8/14		Mean
				min	METs·h	min	METs·h	min	METs·h	min	METs·h	min	METs·h	min	METs·h	METs·h
Exercise	Yoga	02160	4.0	30	2.0	30	2.0	30	2.0	30	2.0	30	2.0	30	2.0	2.0
	Team practice	15560	8.3	150	20.8	60	8.3			300	41.5					23.5
	Stretch	02101	2.3	30	1.2	30	1.2	30	1.2	30	1.2	150	5.8	30	1.2	1.9
	Unit training	15560	8.3			120	16.6					60	8.3			12.5
	Skill training	15562	6.3					60	6.3			60	6.3			6.3
	Core exercise	02160	4.0			30	2.0					30	2.0			2.0
	Weight training	02050	6.0	180	18.0			60	6.0	120	12.0					12.0
	Strength and Fitness	02050	6.0													
	Fitness	02040	8.0													
	Cap Run	12010	6.0											60	6.0	6.0
	Pool	18367	2.5	90	3.8											3.8
	Game	15560	8.3					80	11.1					80	11.1	11.1
	Personal Training	02052	5.0	60	5.0	60	5.0	120	10.0	60	5.0	60	5.0	120	10.0	6.7
	Physical activity	METS·h			50.7		35.1		36.5		61.7		29.4		30.2	40.6
	Energy expenditure	kcal/kg			53.2		36.8		38.3		64.7		30.8		31.7	42.6
Activity other than exercise	Recreation	07023	1.8	240	7.2	450	13.5	400	12.0	240	7.2	390	11.7	460	13.8	10.9
	Meal	07022	1.5	180	4.5	180	4.5	180	4.5	180	4.5	180	4.5	180	4.5	4.5
	Sleep	07030	1.0	480	8.0	480	8.0	480	8.0	480	8.0	480	8.0	480	8.0	8.0
	Physical activity	METs·h			19.7		26		24.5		19.7		24.2		26.3	23.4
	Energy expenditure	kcal/kg			20.7		27.3		25.7		20.7		25.4		27.6	24.6
Total	Physical activity	METs·h			70.4		61.1		61.0		81.4		53.6		56.5	64.0
	Energy expenditure	kcal/kg			73.9		64.1		64.1		85.4		56.2		59.3	67.2

(**B**) Backs

	Category	CODE	METs	8/9		8/10		8/11		8/12		8/13		8/14		Mean
				min	METs·h	min	METs·h	min	METs·h	min	METs·h	min	METs·h	min	METs·h	METs·h

Exercise	Yoga	02160	4	30	2			30	2	30	2	30	2	30	2	2.0
	Team practice	15560	8.3	150	20.75	60	8.3			150	20.75	150	20.75			17.6
	Stretch	02101	2.3	30	1.15	30	1.15	30	1.15	30	1.15	30	1.15	30	1.15	1.2
	Unit training	15560	8.3	150	20.75	120	16.6			120	16.6	90	12.45			16.6
	Skill training	15562	6.3	150	15.75			60	6.3			60	6.3			9.5
	Core exercise	02160	4			30	2									2.0
	Weight training	02050	6			120	12									12.0
	Strength and Fitness	02050	6					60	6							6.0
	Fitness	02040	8							150	20					20.0
	Cap Run	12010	6											60	6	6.0
	Pool	18367	2.5													
	Game	15560	8.3					80	11.0667					80	11.0667	11.1
	Personal Training	02052	5	60	5	60	5	120	10	60	5	60	5	120	10	6.7
	Physical activity	METS·h			65.4		45.1		36.5		65.5		47.7		30.2	48.4
	Energy expenditure	kcal/kg			68.7		47.3		38.3		68.8		50.0		31.7	50.8
Activity other than exercise	Recreation	07023	1.8	210	6.3	360	10.8	400	12	240	7.2	360	10.8	460	13.8	
	Meal	07022	1.5	180	4.5	180	4.5	180	4.5	180	4.5	180	4.5	180	4.5	
	Sleep	07030	1	480	8	480	8	480	8	480	8	480	8	480	8	
	Physical activity	METs·h			18.8		23.3		24.5		19.7		23.3		26.3	22.7
	Energy expenditure	kcal/kg			19.7		24.5		25.7		20.7		24.5		27.6	23.8
Total	Physical activity	METs·h			84.2		68.4		61.0		85.2		71.0		56.5	71.0
	Energy expenditure	kcal/kg			88.4		71.8		64.1		89.5		74.5		59.3	74.6
